# Development and characterization of microsatellite markers in the African forest elephant (*Loxodonta cyclotis*)

**DOI:** 10.1186/s13104-016-2167-3

**Published:** 2016-07-26

**Authors:** Natalie A. Gugala, Yasuko Ishida, Nicholas J. Georgiadis, Alfred L. Roca

**Affiliations:** 1 Department of Animal Sciences, University of Illinois at Urbana-Champaign, Urbana, IL 61801 USA; 2 Puget Sound Institute, University of Washington, Tacoma, WA 98402 USA; 3 The Institute for Genomic Biology, University of Illinois at Urbana-Champaign, Urbana, IL 61801 USA

**Keywords:** Conservation genetics, Lope National Park, Short tandem repeats

## Abstract

**Background:**

African elephants comprise two species, the savanna elephant (*Loxodonta africana*) and the forest elephant (*L. cyclotis*), which are distinct morphologically and genetically. Forest elephants are seriously threatened by poaching for meat and ivory, and by habitat destruction. However, microsatellite markers have thus far been developed only in African savanna elephants and Asian elephants, *Elephas maximus*. The application of microsatellite markers across deeply divergent lineages may produce irregular patterns such as large indels or null alleles. Thus we developed novel microsatellite markers using DNA from two African forest elephants.

**Findings:**

One hundred microsatellite loci were identified in next generation shotgun sequences from two African forest elephants, of which 53 were considered suitable for testing. Twenty-three microsatellite markers successfully amplified elephant DNA without amplifying human DNA; these were further characterized in 15 individuals from Lope National Park, Gabon. Three of the markers were monomorphic and four of them carried only two alleles. The remaining sixteen polymorphic loci carried from 3 to 8 alleles, with observed heterozygosity ranging from 0.27 to 0.87, expected heterozygosity from 0.40 to 0.86, and the Shannon diversity index from 0.73 to 1.86. Linkage disequilibrium was not detected between loci, and no locus deviated from Hardy–Weinberg equilibrium.

**Conclusions:**

The markers developed in this study will be useful for genetic analyses of the African forest elephant and contribute to their conservation and management.

**Electronic supplementary material:**

The online version of this article (doi:10.1186/s13104-016-2167-3) contains supplementary material, which is available to authorized users.

## Findings

Among African elephants, genetic studies have established that savanna elephants, *Loxodonta africana*, and forest elephants, *Loxodonta cyclotis*, are morphologically distinct [1] and comprise deeply divergent lineages separated by 4–7 million years of evolution (e.g. [2, 3]). Forest elephants have been extirpated or reduced to critically low densities across much of their former range, and they remain under serious threat from poaching for meat and ivory, and from habitat destruction [4]. Microsatellite marker studies have thus far been developed only in African savanna elephants and Asian elephants, *Elephas maximus*. The application of microsatellite markers across such deeply divergent lineages may produce null alleles or irregular patterns [5]. For example, some microsatellite markers developed in savanna elephants show an allele size range in forest elephants that suggests the presence of large indels [6]. Likewise, of ten microsatellite loci developed in domestic cats (*Felis catus*) that were later tested in pumas (*Puma concolor*), six loci showed differences in the structure of repeat units and many alleles reflected size homoplasies [5]. In this study, we developed novel microsatellite markers using DNA from two African forest elephants, and tested and characterized the markers using high quality DNA extracted from tissue samples of 15 forest elephants from Lope National Park, Gabon.

This study was conducted under the University of Illinois Institutional Animal Care, and Use Committee approved protocol number 12040. Samples were collected in full compliance with required Convention on International Trade in Endangered Species of Wild Fauna and Flora and other institutional permits.

DNA was extracted as previously described [7]. Genomic DNA samples from two forest elephants, SL0001 from Sierra Leone (West African Guinean Forest) and LO3502 from Lope National Park, Gabon (Central African Congolian Forest), were sequenced on 1/16th of a PicoTiterPlate (PTP) (1/8 PTP total) on the Roche 454 GS FLX+ platform at the UIUC high-throughput sequencing and genotyping unit. The whole genome sequence data was pooled together and MSATCOMMANDER 1.0.8 [8] was run to identify microsatellite repeat motifs by screening the sequences for di-, tri-, tetra- and penta- nucleotide motifs, with a minimum of 8 repeats each. MSATCOMMANDER interfaces with PRIMER 3 software [9], and was modified to allow a minimum length of 18 bp of flanking DNA between the microsatellite repeat and the primer sequences [10, 11]. Primers were designed to amplify a target region of 150 bp or less, inclusive of the two primer lengths, so that they would be appropriate for use with degraded DNA from dung or from other samples with low quality DNA. Default settings were otherwise used for MSATCOMMANDER [8]: optimal primer length of 20 bp (minimum 18 to the maximum 22 bp), optimal melting temperature of 60 °C (range of 58–62 °C).

To preclude the targeting of repetitive regions, the following steps were taken. First, a blast search was performed using each primer sequence as query against the GS FLX reads, using a Perl script to identify primers that would target repetitive regions. Second, NCBI BLAST (http://www.blast.ncbi.nlm.nih.gov/Blast.cgi) was used to search each sequence of the locus against the savanna elephant (*L. africana)* nonredundant database. The short tandem repeats of the sequences were masked using RepeatMasker (http://www.repeatmasker.org) before conducting the Blast search. Third, the software In-Silico PCR on the UCSC genome browser (https://www.genome.ucsc.edu/cgi-bin/hgPcr) was used to query oligonucleotide sequences against the human genome (GRCH37/hg19 assembly) to ensure that primers would not target human DNA, since human DNA contamination may be a concern with highly degraded DNA samples. Forest elephant microsatellite loci that were found to be in repetitive regions or to have high similarity to human DNA sequences were removed from further consideration.

In total, 53 of the first 100 microsatellite potential primer pairs identified using MSATCOMMANDER 1.0.8 [8] passed these criteria. These 53 potential primer pairs were each tested with DNA extracted from two African forest elephants, as well as on DNA from two African savanna elephants, with human DNA and water used as controls. At 18 microsatellite loci, alternative primers were subsequently designed and tested in an attempt to reduce artefactual shadow bands or to improve amplification success; while at two loci new primers were designed and tested after the originals were found to amplify human DNA in the negative control. We verified that 23 of 53 microsatellite loci produced amplicons in both elephant species without amplifying human DNA. We characterised these 23 microsatellite loci using the 15 forest elephant samples from Lope National Park, Gabon (Table 1).

**Table 1:** Characterization of 23 microsatellite markers in 15 forest elephants from Lope National Park, Gabon

Locus	Forward primer sequence (5′–3′)	Reverse primer sequence (5′–3′)	*n*	No. of alleles	*Ho*	*He*	*I*	Allele size range (bp)	Repeat motif	GenBank accession no.
Lcy-M4	Lcy-M4-F: GGAGAGAGTCTGTGCACCTC	Lcy-M4-R: ATGAGTGTGTGCATGGAACG	15	4	0.27	0.43	0.82	122–128	(AC)_8_	KU947083
Lcy-M6	Lcy-M6-F: ACGGCAATTTAAGATGGAGCC	Lcy-M6-R: CGGTCATTTGCAACACTGTG	15	5	0.80	0.72	1.33	130–140	(AC)_9_	KU947085
Lcy-M8	Lcy-M8-F: TTGAAATTCAGATCAGCGTGTG	Lcy-M8-R: CAGGGCTTTAGTTCACGCTC	15	6	0.67	0.72	1.42	125–149	(TG)_8_	KU947086
Lcy-M16	Lcy-M16-F: CTGATCACTTCTTAGCGGTGTC	Lcy-M16-R: GGTGTTCCTGGACATCTGCC	15	5	0.67	0.69	1.26	133–155	(AC)_14_	KU947088
Lcy-M17	Lcy-M17-F: GGAGCTCAGGAAATACACAGC	Lcy-M17-R: TGATCGCCTCTGTTTCGTTC	15	4	0.47	0.40	0.73	146–156	(TG)_11_	KU947089
Lcy-M23	Lcy-M23.2-F: TGAAGGCGTCTCTGTTATTGC	Lcy-M23.2-R: GAAGCCAAGCAAATGGGATA	15	4	0.60	0.46	0.87	160–168	(AC)_12_	KU947092
Lcy-M24	Lcy-M24.2-F: GATGACTCTGGCTCCTGGAT	Lcy-M24.2-R: CGCCCTGACTGGTCTTACTC	15	5	0.80	0.76	1.43	114–132	(TG)_16_	KU947093
Lcy-M26	Lcy-M26-F: CAACCAAGTTCTGCCTGCTG	Lcy-M26-R: CGTGGTCTCTTTGCTGTCAC	15	7	0.80	0.86	1.85	148–162	(AC)_10_	KU947094
Lcy-M27	Lcy-M27.2-F: TCCTTGATGTGGTTTCAGTCA	Lcy-M27.2-R: GGTAGGTTGGCTATGTTTCTTG	15	3	0.53	0.48	0.80	123–131	(AC)_9_	KU947095
Lcy-M29	Lcy-M29-F: AGCCTCTTTCTTTCCGTTGC	Lcy-M29-R: AGATCCTCAGGGTAATTTGCAC	15	5	0.47	0.67	1.22	160–170	(TG)_8_	KU947096
Lcy-M30	Lcy-M30-F: CACTACGCCAACAGGTTTCC	Lcy-M30-R: TGTCCCATAGTGTCACCGTG	15	3	0.60	0.52	0.87	150–156	(AC)_8_	KU947097
Lcy-M40	Lcy-M40-F: TTCAAGTACCTGCTGTCACTG	Lcy-M40-R: TTCTGTCCAAGTAGCTTACCTG	15	3	0.47	0.57	0.88	160–166	(AC)_9_	KU947099
Lcy-M43	Lcy-M43.2-F: TCCTCACTGCCACAACCAT	Lcy-M43.2-R: GGGTCTCTTCCCATCATCAG	15	8	0.87	0.81	1.83	135–155	(AC)_26_	KU947101
Lcy-M44	Lcy-M44.2-F: GGCCTGATATAGCCCTTGT	Lcy-M44.2-R: TCCTGCTGTACTTGAATTTCTGA	15	6	0.80	0.77	1.57	128–144	(AC)_11_	KU947102
Lcy-M45	Lcy-M45.3-F: CAGCTATTCTATCCTCACAAGTCCT	Lcy-M45.3-R: TGTGAATGGAGAGTTTTCTCTGA	15	8	0.87	0.85	1.86	123–147	(AC)_18_	KU947103
Lcy-M50	Lcy-M50.2-F: CCCAGGACCAGCATAGTGAT	Lcy-M50.2-R: TGGAATAAATCAAGAGTAAGAATTCAC	15	4	0.60	0.56	1.02	137–147	(AG)_11_	KU947104
Lcy-M15^a^	Lcy-M15-F: AATCAGGCAGCTAACAACGG	Lcy-M15-R: CAGCCCTTCACAGAAAGTCC	15	2	0.07	0.07	0.15	148–150	(TG)_9_	KU947087
Lcy-M20^a^	Lcy-M20.2-F: GTCTTCCAAAGCACCCTCTG	Lcy-M20.2-R: AATAGACGGGAGAGGGGAGT	15	2	0.47	0.36	0.54	156–158	(AC)_10_	KU947090
Lcy-M22^a^	Lcy-M22-F: CTTGAGCCTGCTGTATGTGC	Lcy-M22-R: CAGAAGCTGGATGGTCAAGC	15	2	0.13	0.13	0.25	155–157	(TG)_10_	KU947091
Lcy-M39^a^	Lcy-M39.2-F: CATGGTGACTGCTGCATTG	Lcy-M39.2-R: TGTTGCTTGTTCTGTCCCTAGA	15	2	0.07	0.28	0.45	157–163	(AGC)_10_	KU947098
Lcy-M5^a^	Lcy-M5-F: GTAGTTGCCTCAAGTTCCAGC	Lcy-M5-R: CTCCAACACTCCTCCCTCTG	15	1	0.00	0.00	0.00	121	(AT)_10_	KU947084
Lcy-M42^a^	Lcy-M42.2-F: AAGGCATATGCAGGAAGCTG	Lcy-M42.2-R: AACTGTGCTCCAGGGTCACT	14	1	0.00	0.00	0.00	120	(AC)_19_	KU947100
Lcy-M52^a^	Lcy-M52-F: CCGTAAGCTAATCCTCCTGC	Lcy-M52-R: ATTTGCCTTTGTTCCTGCCG	15	1	0.00	0.00	0.00	168	(AC)_8_	KU947105

An M13 forward tail (TGTAAAACGACGGCCAGT) that was at the 5′ end of each forward primer is not shown above

PCR algorithms are detailed in Additional file 1

Allele sizes shown here include both forward and reverse primer lengths including the M13 forward tail length

*Ho* and *He* represent observed and expected heterozygosity, respectively, *I* represents Shannon’s diversity index

^a^Linkage disequilibrium and deviation from Hardy–Weinberg equilibrium were not examined due to low genetic diversity

All forward primers included the M13 forward sequence (TGTAAAACGACGGCCAGT) at the 5′ end. A FAM- or VIC- fluorescent labeled M13 forward primer was included in the reaction to label the PCR amplicon [12], along with a conventional reverse primer. The PCR mix consisted of 1X PCR buffer II (Life Technologies, Carlsbad, CA, USA), 2 mM MgCl_2_, 200 μM of each dNTP (Life Technologies) with 0.04 units/μl final concentration of AmpliTaq Gold DNA Polymerase (Life Technologies) along with 1.2 µl of primer mix. A detailed protocol describing the components of the PCR mix is provided in the Additional file 1.

The PCR cycling program for all but one of the primer pairs was designated “touchdown 50” and is described in the Additional file 1. The locus Lcy-M45 produced a high degree of artefactual shadow bands, so the PCR cycling program was modified and the final annealing temperature was kept higher to reduce background noise. This alternative was designated “touchdown 56” and is also detailed in the Additional file 1. PCR amplicons were run on a 2 % agarose gel with ethidium bromide and examined under UV light. The remaining amplicon from two different loci that were labeled with different fluorescent dyes were mixed, then diluted depending on the intensity of the image on the agarose gel photo, followed by analyses on an ABI 3730XL capillary sequencer at the University of Illinois at Urbana-Champaign High-Throughput Sequencing and Genotyping Unit. The software Genemapper Version 3.7 (Life Technologies) was used to call and bin alleles. Genotyping was conducted independently by two individuals to ensure consistency of calls.

MS Tool v3 [13], Arlequin version 3.5.1.3 [14], and GenAlEx 6.5 [15] were used to calculate expected heterozygosity (*He*) and observed heterozygosity (*Ho*); the estimates were confirmed as consistent among the software packages. GenAlEx 6.5 [15] was also used to calculate Shannon’s diversity index (*I*) and to make allele histograms for each locus (Additional file 1). Shannon’s diversity index is used to quantify biological diversity and accounts for both abundance and evenness of the variation present. Shannon’s diversity index uses allele frequencies and quantifies the informativeness of the markers, with higher values for more informative markers, and a value of 0 for monomorphic markers [16]. Microsatellite data were tested for deviation from Hardy–Weinberg equilibrium (HWE) and for linkage disequilibrium (LD) using Genepop 4.2 [17]. A Markov chain algorithm was used to test for HWE using 10,000 dememorization steps, 200 batches and 1000 iterations per batch. LD was tested using 10,000 dememorization steps, 100 batches and 1000 iterations per batch for each pairwise comparison between loci.

The 23 primer pairs successfully amplified microsatellite products in all 15 DNA samples from Lope (Table 1). Microsatellite markers Lcy-M5, Lcy-M42, and Lcy-M52 were monomorphic and Lcy-M15, Lcy-M20, Lcy-M22, and Lcy-M39 carried only two alleles in the Lope samples and were removed from further consideration. At the remaining 16 loci, the number of alleles ranged from 3 to 8 with an average of 5.00 ± 0.41 (SE). The average observed heterozygosity across the 16 loci was 0.64 ± 0.04 (SE), with the highest value being 0.87. The average expected heterozygosity across the 16 loci was 0.63 ± 0.04 (SE), with the highest value being 0.86. The average Shannon diversity index was 1.23 ± 0.10 (SE), with a range from 0.73 to 1.86. There was no significant linkage disequilibrium between markers after Bonferroni correction (*p* > 0.0004). Among 16 markers, none deviated from *HWE* after Bonferroni correction (*p* > 0.003).

Of the 53 novel microsatellite markers developed in forest elephants, 23 loci were tested and characterized. Sixteen of 23 markers displayed more than 2 alleles and are recommended for future use. These microsatellite markers will allow assessment of the genetic diversity and structure of forest elephant populations, which would aid in their conservation and management.

## Additional file

10.1186/s13104-016-2167-3 Additional material.

## Abbreviations

*He*: expected heterozygosity
*Ho*: observed heterozygosity
HWE: Hardy–Weinberg equilibrium
LD: linkage disequilibrium
LO: Lope National Park, Gabon
PTP: picotiterplate
SE: standard error
SL: Sierra Leone

## References

[1] Grubb P, Groves CP, Dudley JP, Shoshani J (2000). Living African elephants belong to two species: *Loxodonta africana* (Blumenbach, 1797) and *Loxodonta cyclotis* (Matschie, 1900). Elephant.

[2] Brandt AL, Ishida Y, Georgiadis NJ, Roca AL (2012). Forest elephant mitochondrial genomes reveal that elephantid diversification in Africa tracked climate transitions. Mol Ecol.

[3] Roca AL, Ishida Y, Brandt AL, Benjamin NR, Zhao K, Georgiadis NJ (2015). Elephant natural history: a genomic perspective. Ann Rev Anim Biosci.

[4] Maisels F, Strindberg S, Blake S, Wittemyer G, Hart J, Williamson EA, Aba’a R, Abitsi G, Ambahe RD, Amsini F (2013). Devastating decline of forest elephants in central Africa. PLoS ONE.

[5] Culver M, Menotti-Raymond MA, O’Brien SJ (2001). Patterns of size homoplasy at 10 microsatellite loci in pumas (*Puma concolor*). Mol Biol Evol.

[6] Comstock KE, Georgiadis N, Pecon-Slattery J, Roca AL, Ostrander EA, O’Brien SJ, Wasser SK (2002). Patterns of molecular genetic variation among African elephant populations. Mol Ecol.

[7] Roca AL, Georgiadis N, Pecon-Slattery J, O’Brien SJ (2001). Genetic evidence for two species of elephant in Africa. Science.

[8] Faircloth BC (2008). Msatcommander: detection of microsatellite repeat arrays and automated, locus-specific primer design. Mol Ecol Resour.

[9] Rozen S, Skaletsky H (2000). Primer3 on the WWW for general users and for biologist programmers. Methods Mol Biol.

[10] Brandt JR, Van Coeverden de Groot PJ, Zhao K, Dyck MG, Boag PT, Roca AL (2014). Development of nineteen polymorphic microsatellite loci in the threatened polar bear (*Ursus maritimus*) using next generation sequencing. Conserv Genet Resour.

[11] Ruiz-Rodriguez C, Ishida Y, Greenwood A, Roca A. Development of 14 microsatellite markers in the Queensland koala (*Phascolarctos cinereus adustus*) using next generation sequencing technology. Conserv Genet Resour. 2014: 1–3. 10.1007/s12686-013-0115-2 PMC4109682 25067980

[12] Boutin-Ganache I, Raposo M, Raymond M, Deschepper CF (2001). M13-tailed primers improve the readability and usability of microsatellite analyses performed with two different allele-sizing methods. Biotechniques..

[13] Parks SDE. Trypanotolerance in West African cattle and the population genetic effects of selection. Ph.D thesis. University of Dublin; 2001.

[14] Excoffier L, Lischer HE (2010). Arlequin suite ver 3.5: a new series of programs to perform population genetics analyses under Linux and Windows. Mol Ecol Resour..

[15] Peakall R, Smouse PE (2012). GenAlEx 6.5: genetic analysis in Excel. Population genetic software for teaching and research–an update. Bioinformatics.

[16] Sherwin WB, Jabot F, Rush R, Rossetto M (2006). Measurement of biological information with applications from genes to landscapes. Mol Ecol.

[17] Rousset F (2008). Genepop’007: a complete re-implementation of the genepop software for Windows and Linux. Mol Ecol Resour.

