# Supplementary material for: Development and characterization of microsatellite markers in the African forest elephant (Loxodonta cyclotis)
Source: BMC Res Notes. 2016 Jul 26;9:364. doi: 10.1186/s13104-016-2167-3 (PMC4960834; doi:10.1186/s13104-016-2167-3)
Supplement: Supplementary file 1 — 10.1186/s13104-016-2167-3 Additional material. [file 13104_2016_2167_MOESM1_ESM.pdf]

Additional file 1

Development and characterization of microsatellite markers in the African forest elephant (*Loxodonta cyclotis*)

Natalie A. Gugala[1, §], Yasuko Ishida[1, §,*], Nicholas J. Georgiadis[2], Alfred L. Roca[1,3,*]

1 Department of Animal Sciences, University of Illinois at Urbana-Champaign, Urbana, IL 61801, USA
2 Puget Sound Institute, University of Washington, Tacoma, WA 98402, USA
3 The Institute for Genomic Biology, University of Illinois at Urbana-Champaign, Urbana, IL 61801, USA

[§]These authors contributed equally

*Corresponding authors:
Yasuko Ishida, Ph.D. (yishida@illinois.edu), Alfred L. Roca, Ph.D. (roca@illinois.edu)

Supplementary protocols

Details of the PCR setup and PCR algorithm

**Primer mix**	volume (μl)
20 μM reverse primer	100
20 μM M13 tailed forward primer	7.5
100 μM M13 fluorescent labeled primer	20
TLE (10 mM Tris-HCl, 0.1 mM EDTA) or mili-Q filtered water	107.5

**PCR components**	volume (μl) for 1 sample
Sterile mili-Q filtered water	6.02[a]
10X PCR Buffer II[b]	1.00
dNTP Mix (10mM)[c]	0.80
$MgCl_2$ (25mM)[b]	0.80
AmpliTaq Gold DNA Polymerase[b]	0.08
Primer mix (follow above recipe)	0.80
Template DNA	0.50[a]
TOTAL volume	10.00

[a]Depending on the quality or quantity of DNA, the volumes of DNA and of water used may need to be adjusted, with the total reaction volume remaining as 10 μl.
[b]AmpliTaq Gold DNA Polymerase with Buffer II and $MgCl_2$ solution (ABI, N8080249).
[c]2.5mM of each dNTP (dATP, dCTP, dGTP and dTTP) blend (ABI, N8080260).
Please refer Ishida et al. (Ishida, et al. 2012) for DNA extracted from dung samples.

PCR algorithms

1. Touchdown 50
10 min at 95˚C
2 cycles of 15 sec at 95˚C, 30 sec at 60˚C, 45 sec at 72˚C
2 cycles of 15 sec at 95˚C, 30 sec at 58˚C, 45 sec at 72˚C
2 cycles of 15 sec at 95˚C, 30 sec at 56˚C, 45 sec at 72˚C
2 cycles of 15 sec at 95˚C, 30 sec at 54˚C, 45 sec at 72˚C
2 cycles of 15 sec at 95˚C, 30 sec at 52˚C, 45 sec at 72˚C
30 cycles of 15 sec at 95˚C, 30 sec at 50˚C, 45 sec at 72˚C
30 min final extension at 72˚C
Hold at 4˚C

2. Touchdown 56
10 min at 95˚C
2 cycles of 15 sec at 95˚C, 30 sec at 60˚C, 45 sec at 72˚C
2 cycles of 15 sec at 95˚C, 30 sec at 58˚C, 45 sec at 72˚C
36 cycles of 15 sec at 95˚C, 30 sec at 56˚C, 45 sec at 72˚C
30 min final extension at 72˚C
Hold at 4˚C

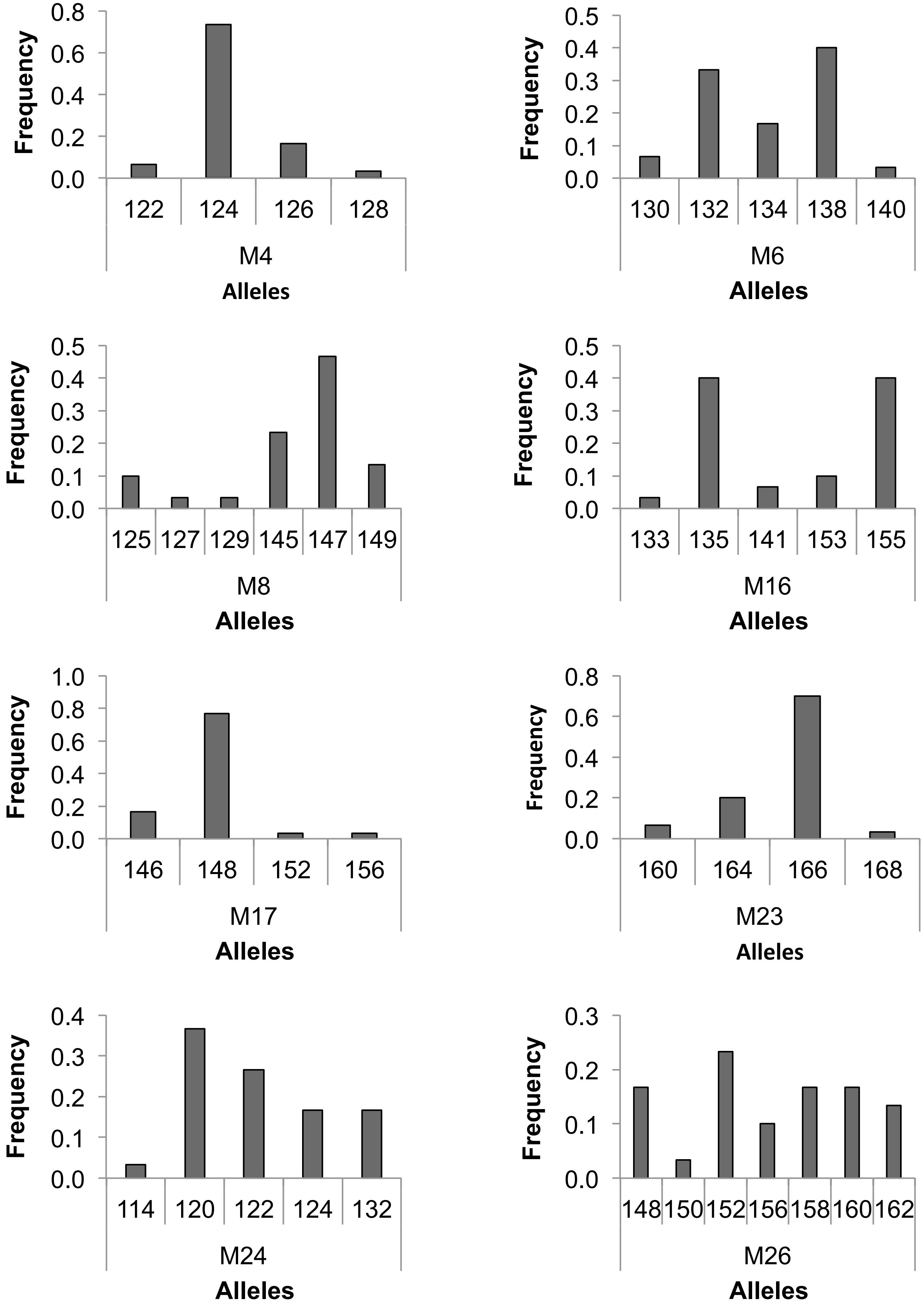

Figure S1 (continues next page).

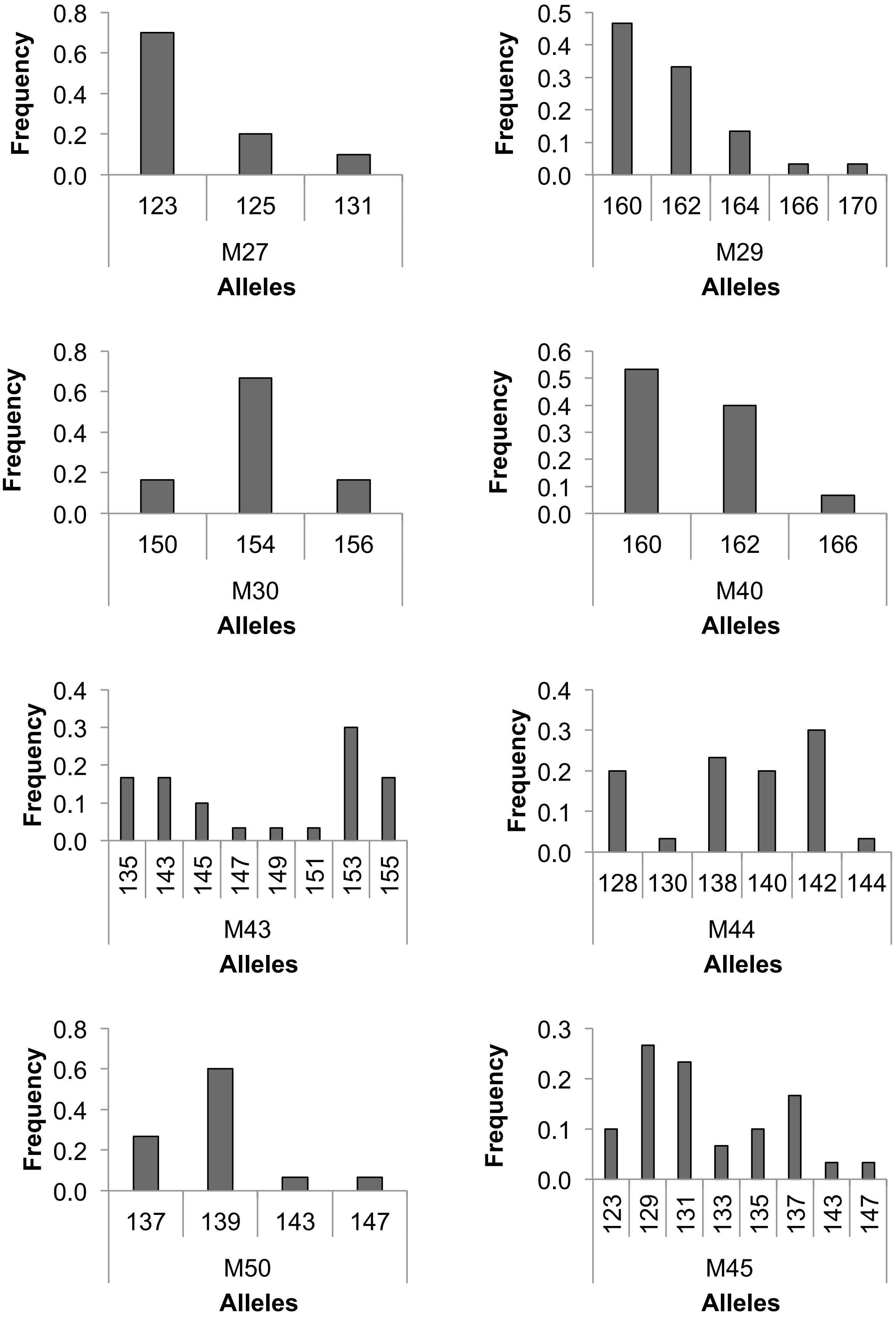

Figure S1 (continues next page).

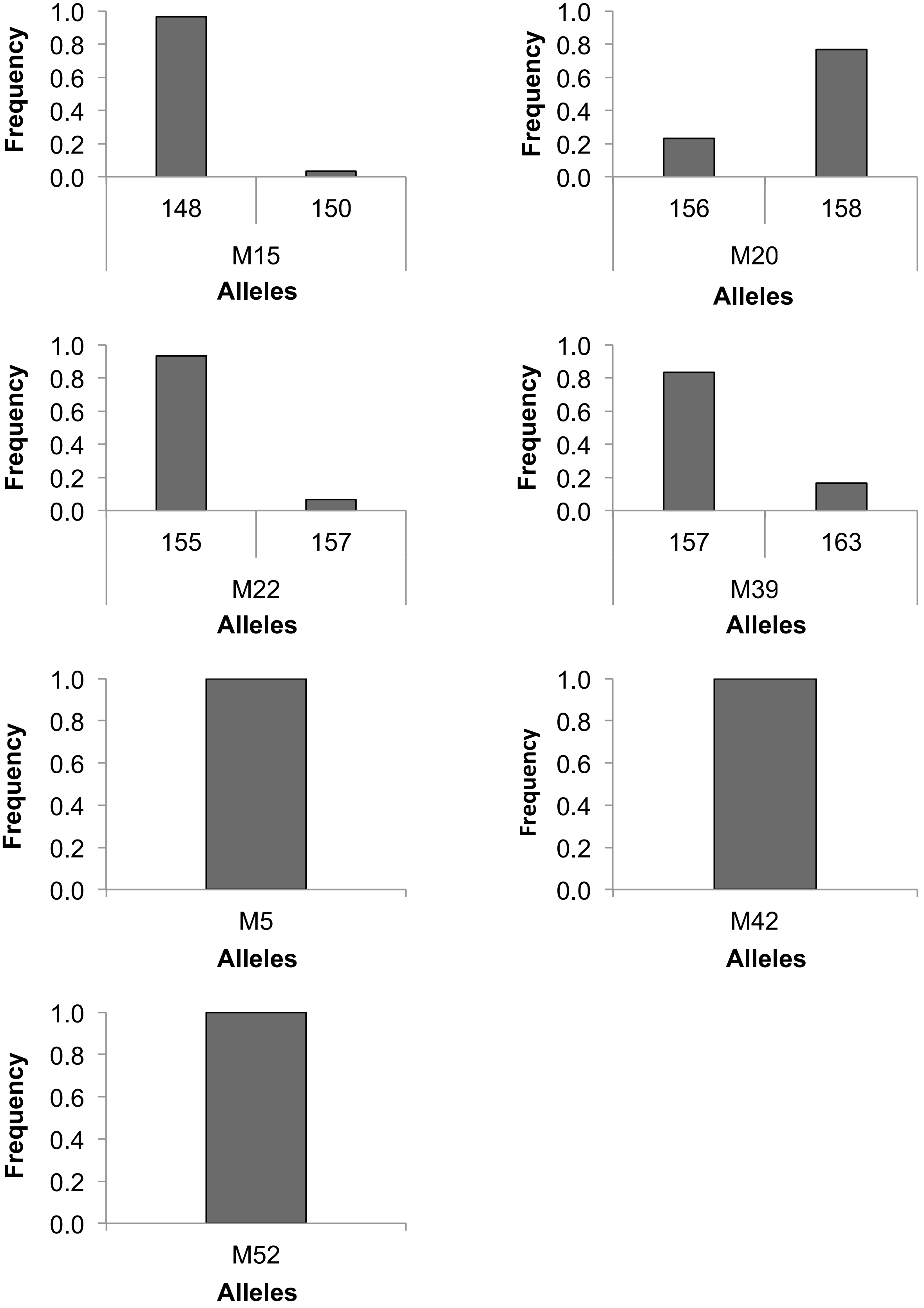

Figure S1. Frequency distribution of alleles at 23 microsatellite loci across 15 forest elephants from Lope National Park, Gabon. Lcy-M5, -M42, and -M52 were monomorphic with only one allele and Lcy-M15, M20, -M22, -M39 carried two alleles. The rest of the loci were polymorphic, ranging from 3 to 8 alleles per locus.

References

1. Ishida Y, Demeke Y, van Coeverden de Groot P, Georgiadis N, Leggett KA, Fox V, Roca A: **Short amplicon microsatellite markers for low quality elephant DNA**. *Conservation Genetics Resources* 2012, **4**(2):491-494.
